# Crystal structure of 2-oxopyrrolidin-3-yl 4-(2-phenyl­diazen-1-yl)benzoate

**DOI:** 10.1107/S205698901800333X

**Published:** 2018-03-06

**Authors:** Igor Elkin, Thierry Maris, Alexandre Melkoumov, Patrice Hildgen, Xavier Banquy, Grégoire Leclair, Christopher Barrett

**Affiliations:** a Otto Maass Chemistry Building, Office 430, Chemistry Department, McGill University, 801 Sherbrooke St. W., Montreal, Quebec, Canada, H3A 0B8; b Department of Chemistry, Université de Montréal, 2900 Edouard-Montpetit Blvd., Montreal, Quebec, Canada, H3C 3J7; c Faculty of Pharmacy, Université de Montréal, 2900 Edouard-Montpetit Blvd., Montreal, Quebec, Canada, H3C 3J7

**Keywords:** crystal structure, azo­benzene, 2-pyrrolidone, cyclic γ-amino­butyric acid derivative, GABA, racetam

## Abstract

In the title compound, C_17_H_15_N_3_O_3_, the plane of the pyrrolidone ring is inclined at an angle of 59.791 (2)° to that of the azo­benzene segment, which adopts a configuration close to planar. In the crystal, mol­ecules are oriented pairwise by (2-oxopyrrolidin-3-yl)­oxy moieties at an angle of 76.257 (3)°, linked by hydrogen bonds and π-stacking inter­actions, forming zigzag supra­molecular chains parallel to [010] further linked *via* additional C—H⋯π inter­actions.

## Abstract 2

The crystal preparation and structure of a cyclic derivative of γ-amino­butyric acid, GABA, is reported.

## Chemical context   

Cyclic derivatives of γ-amino­butyric acid, GABA, are still constituting a very promising avenue for developing new drug-mol­ecules for improving neuronal, vascular and general cognitive functions (Malykh *et al.*, 2010 ▸). In this context, the goal of the present study was to obtain crystals and to characterize the mol­ecular structure of a new representative of the cyclic-GABA family (racetams), 2-oxopyrrolidin-3-yl 4-(2-phenyl­diazen-1-yl)benzoate.
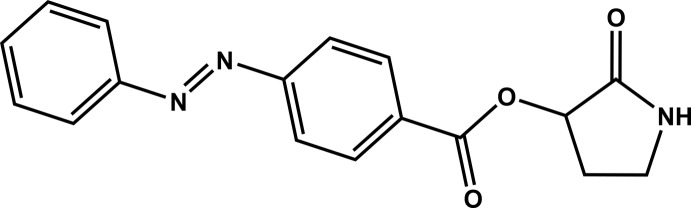


## Structural commentary   

**Figure 1:**
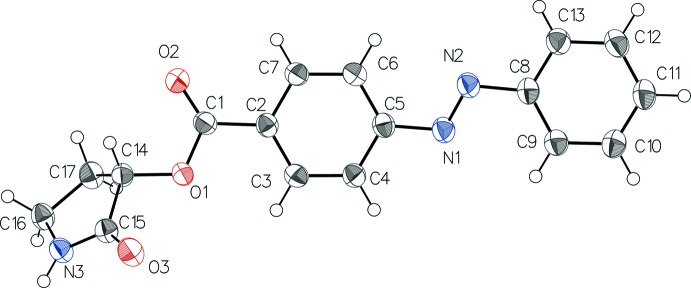
The mol­ecular structure of the title compound, with displacement ellipsoids drawn at the 50% probability level. H atoms are shown as small spheres of arbitrary radius.

The mol­ecular structure of the title compound (Fig. 1 ▸) comprises the expected 4-phenyl­azobenzoyl and (2-oxopyrrolidin-3-yl)­oxy segments linked by the carb­oxy­ester bond —C1(=O2)—O1. The phenyl­azobenzoyl segment comprises two aromatic rings, one of which is linked at its *para*-position to the carbonyl C8 atom, and exhibits the more stable *trans* configuration to the azo group formed by N1—N2 atoms with a distance of 1.251 (5) Å. No residual peaks are observed around the N=N double bond as for pure azo­benzene where such peaks are observed due to a dynamic pedal-like motion orientational disorder (Harada *et al.*, 2004 ▸). The angle between the two phenyl rings is 4.29 (13)° and is consistent with a slight deviation of the system from the ideal planarity. Geometry parameters of the 3-oxy-substituted 2-pyrrolidone segment are close to known data (Clark *et al.*, 2006 ▸), with a typical deviation from planarity for the non-aromatic system as shown by the torsion angles C15—C14—C17—C16 of 22.2 (4)° and C17—C14—C15—N3 of −16.3 (4)°. The Cremer & Pople puckering parameters of the five-membered ring are *Q* = 0.222 (4) Å and φ = 279.4 (11)° conforming to an envelope on C17 (Boeyens, 1978 ▸; Cremer & Pople, 1975 ▸).

## Supra­molecular features   

**Figure 2:**
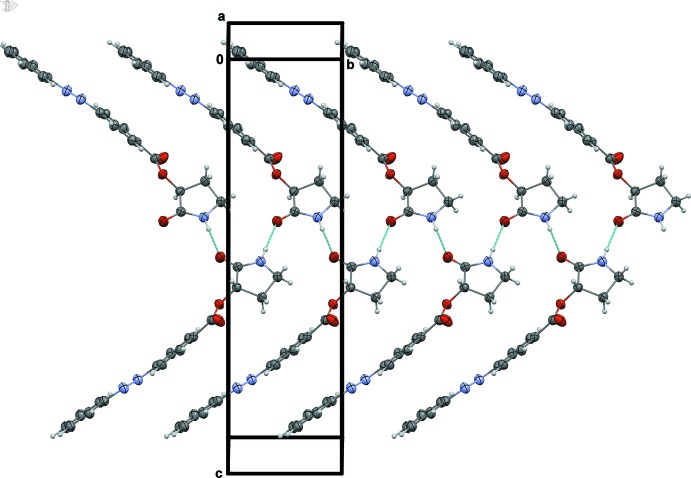
A partial view of the crystal packing of the title compound. Hydrogen bonds are shown as dashed lines (see Table 1 ▸).

The packing of the title mol­ecules in the crystal (Fig. 2 ▸) is mainly determined by the presence of (2-oxopyrrolidin-3-yl)­oxy moieties inter­acting with each other pairwise, by forming hydrogen bonds between secondary amine and carbonyl groups (Table 1 ▸), similarly to other 3-oxy-substituted 2-pyrrolidone derivatives (Clark *et al.*, 2006 ▸). This inter­action together with a π–π inter­action between the two different phenyl rings from the azo­benzene moieties of adjacent mol­ecules [distance between centroids of 3.934 (2) Å] define a chain of corrugated mol­ecules running along the *b-*axis direction (Fig. 2 ▸). The inter­actions between these chains proceed through C—H⋯π contacts involving the C8–C13 ring and the terminal atom C11 (Table 1 ▸)

## Database survey   

A search in the Cambridge Structural Database (Version 5.39 with one update, Groom *et al.*, 2016 ▸) returned 101 entries for unsubstituted azo­benzene, including the dynamic disorder study of Harada & Ogawa (2004 ▸); five entries for *O*-*para*-phenyl­azobenzoyl monoesters (Fitjer *et al.*, 1984 ▸; Fujino *et al.*, 2007 ▸; Nakatsuji *et al.*, 2007 ▸, Park *et al.*, 2015 ▸); and only two entries for 3-oxy-substituted 2-pyrrolidone (Clark *et al.*, 2006 ▸).

## Purification and crystallization   

Before recrystallization, 3-oxy(4-phenyl­azobenzo­yl)-2-pyrrolidone was purified by the technique of flash chromatography on silica on Combi Flash Rf 150 (Teledine ISCO, Lincoln, Nebraska, USA) equipped with a SiliaSep (40 g, FLH-R10030B-ISO40) flash-cartridge provided by SiliCycle Inc. (Quebec, QC, Canada), using as eluent the 0–100% gradient of hexa­ne–ethyl acetate, respectively. The purity and structure of the eluate components were confirmed by the LC–MS method on an Agilent Technologies 1260 Infinity LC–MS spectrometer (Santa Clara, CA, US) in ESI positive and negative modes, equipped with an Agilent Poroshell 120 EC–C18 2.7 µm column, using as eluent the 0–100% gradient of solvent mixtures *A* and *B* [where *A*: water–aceto­nitrile (95%–5%) and acetic acid (0.1%); *B*: aceto­nitrile (100%) and acetic acid (0.1%)] at the following conditions: a capillary voltage of ESI source of 3000 V; a vaporizer temperature of 433 K, a nebulization pressure of 60 psig, a dry gas temperature of 573 K, and a gas flow of 5 L min^−1^.

The crystals of the purified product were obtained by the vapor-diffusion method. A solution of 0.05 g of 3-oxy(4-phenyl­azobenzo­yl)-2-pyrrolidone in 1 mL of chloro­form, in a small open container, was placed in a sealed larger container filled with hexane, above the level of the solvent, to give orange needle-shaped crystals.

## Refinement   

Crystal data, data collection and structure refinement details are summarized in Table 2 ▸. H atoms bound to C and N were positioned geometrically with C—H = 0.95–1.00 Å and N—H = 0.88 Å, and refined using a riding model with *U*_iso_(H) = 1.2*U*_eq_(C or N).

## Supplementary Material

Crystal structure: contains datablock(s) I. DOI: 10.1107/S205698901800333X/ff2152sup1.cif

Structure factors: contains datablock(s) I. DOI: 10.1107/S205698901800333X/ff2152Isup2.hkl

Supporting information file. DOI: 10.1107/S205698901800333X/ff2152Isup3.cml

CCDC reference: 1826009

Additional supporting information:  crystallographic information; 3D view; checkCIF report

## Figures and Tables

**Table 1:** Hydrogen-bond geometry (Å, °) *Cg*3 is the centrod of the C8–C13 ring.

*D*—H⋯*A*	*D*—H	H⋯*A*	*D*⋯*A*	*D*—H⋯*A*
N3—H3*A*⋯O3^i^	0.88	1.99	2.868 (4)	175
C11—H11⋯*Cg*3^ii^	0.95	2.76	3.596 (5)	147

Symmetry codes: (i) 
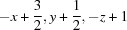
; (ii) 
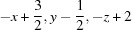
.

**Table 2:** Experimental details

Crystal data	
Chemical formula	C_17_H_15_N_3_O_3_
*M*_r_	309.32
Crystal system, space group	Monoclinic, *C*2
Temperature (K)	150
*a*, *b*, *c* (Å)	10.2069 (3), 6.3761 (2), 23.2265 (7)
β (°)	101.454 (1)
*V* (Å^3^)	1481.48 (8)
*Z*	4
Radiation type	Ga *K*α, λ = 1.34139 Å
μ (mm^−1^)	0.51
Crystal size (mm)	0.38 × 0.09 × 0.06

Data collection	
Diffractometer	Bruker Venture Metaljet
Absorption correction	Multi-scan (*SADABS*; Krause *et al.*, 2015 ▸)
*T*_min_, *T*_max_	0.433, 0.581
No. of measured, independent and observed [*I* > 2σ(*I*)] reflections	21356, 3382, 3014
*R*_int_	0.046
(sin θ/λ)_max_ (Å^−1^)	0.650

Refinement	
*R*[*F* ^2^ > 2σ(*F* ^2^)], *wR*(*F* ^2^), *S*	0.070, 0.201, 1.10
No. of reflections	3382
No. of parameters	210
No. of restraints	1
H-atom treatment	H-atom parameters constrained
Δρ_max_, Δρ_min_ (e Å^−3^)	0.43, −0.26
Absolute structure	Refined as an inversion twin.

Computer programs: *SAINT* and *APEX3* (Bruker, 2016 ▸), *SHELXT* (Sheldrick, 2015*a* ▸), *SHELXL2018* (Sheldrick, 2015*b* ▸), *OLEX2* (Dolomanov *et al.*, 2009 ▸), *Mercury* (Macrae *et al.*, 2008 ▸) and *publCIF* (Westrip, 2010 ▸).

## supplementary crystallographic information

### Crystal data

**Table:**

C_17_H_15_N_3_O_3_	*F*(000) = 648
*M**_r_* = 309.32	*D*_x_ = 1.387 Mg m^−^^3^
Monoclinic, *C*2	Ga *K*α radiation, λ = 1.34139 Å
*a* = 10.2069 (3) Å	Cell parameters from 9959 reflections
*b* = 6.3761 (2) Å	θ = 3.4–60.6°
*c* = 23.2265 (7) Å	µ = 0.51 mm^−^^1^
β = 101.454 (1)°	*T* = 150 K
*V* = 1481.48 (8) Å^3^	Needle, orange
*Z* = 4	0.38 × 0.09 × 0.06 mm

### Data collection

**Table:**

Bruker Venture Metaljet diffractometer	3382 independent reflections
Radiation source: Metal Jet, Gallium Liquid Metal Jet Source	3014 reflections with *I* > 2σ(*I*)
Helios MX Mirror Optics monochromator	*R*_int_ = 0.046
Detector resolution: 10.24 pixels mm^-1^	θ_max_ = 60.7°, θ_min_ = 3.4°
ω and φ scans	*h* = −13→13
Absorption correction: multi-scan (SADABS; Krause *et al.*, 2015)	*k* = −8→8
*T*_min_ = 0.433, *T*_max_ = 0.581	*l* = −30→30
21356 measured reflections	

### Refinement

**Table:**

Refinement on *F*^2^	Hydrogen site location: inferred from neighbouring sites
Least-squares matrix: full	H-atom parameters constrained
*R*[*F*^2^ > 2σ(*F*^2^)] = 0.070	*w* = 1/[σ^2^(*F*_o_^2^) + (0.1386*P*)^2^ + 0.8495*P*] where *P* = (*F*_o_^2^ + 2*F*_c_^2^)/3
*wR*(*F*^2^) = 0.201	(Δ/σ)_max_ < 0.001
*S* = 1.10	Δρ_max_ = 0.43 e Å^−^^3^
3382 reflections	Δρ_min_ = −0.26 e Å^−^^3^
210 parameters	Extinction correction: (SHELXL2018; Sheldrick, 2015b), Fc^*^=kFc[1+0.001xFc^2^λ^3^/sin(2θ)]^-1/4^
1 restraint	Extinction coefficient: 0.0052 (14)
Primary atom site location: dual	Absolute structure: Refined as an inversion twin.

### Special details

**Table:**

Experimental. X-ray crystallographic data for I were collected from a single crystal sample, which was mounted on a loop fiber. Data were collected using a Bruker Venture diffractometer equipped with a Photon 100 CMOS Detector, a Helios MX optics and a Kappa goniometer. The crystal-to-detector distance was 4.0 cm, and the data collection was carried out in 1024 x 1024 pixel mode.
Geometry. All esds (except the esd in the dihedral angle between two l.s. planes) are estimated using the full covariance matrix. The cell esds are taken into account individually in the estimation of esds in distances, angles and torsion angles; correlations between esds in cell parameters are only used when they are defined by crystal symmetry. An approximate (isotropic) treatment of cell esds is used for estimating esds involving l.s. planes.
Refinement. Refined as a 2-component inversion twin.

### Fractional atomic coordinates and isotropic or equivalent isotropic displacement parameters (Å^2^)

**Table:**

	*x*	*y*	*z*	*U*_iso_*/*U*_eq_	
N1	0.6922 (3)	0.2037 (6)	0.83351 (15)	0.0367 (7)	
O1	0.5843 (2)	0.9207 (5)	0.64242 (11)	0.0330 (6)	
C1	0.4923 (4)	0.8542 (6)	0.67255 (17)	0.0329 (8)	
C2	0.5463 (4)	0.6943 (6)	0.71746 (16)	0.0309 (7)	
O2	0.3783 (3)	0.9167 (6)	0.66315 (14)	0.0473 (8)	
N2	0.6075 (3)	0.0794 (5)	0.84632 (15)	0.0367 (7)	
C3	0.6825 (4)	0.6531 (6)	0.73359 (17)	0.0337 (8)	
H3	0.744646	0.733246	0.717269	0.040*	
N3	0.6575 (3)	1.2842 (6)	0.54545 (15)	0.0359 (7)	
H3A	0.708694	1.324852	0.521220	0.043*	
O3	0.6895 (3)	0.9284 (5)	0.53780 (13)	0.0447 (7)	
C4	0.7272 (4)	0.4953 (6)	0.77341 (17)	0.0345 (8)	
H4	0.820379	0.470969	0.785656	0.041*	
C5	0.6365 (4)	0.3719 (6)	0.79569 (16)	0.0327 (8)	
C6	0.4994 (4)	0.4147 (7)	0.78038 (16)	0.0351 (8)	
H6	0.437535	0.333176	0.796487	0.042*	
C7	0.4545 (4)	0.5760 (7)	0.74172 (17)	0.0344 (8)	
H7	0.361682	0.606899	0.731581	0.041*	
C8	0.6650 (4)	−0.0912 (7)	0.88292 (16)	0.0344 (8)	
C9	0.8026 (4)	−0.1300 (6)	0.89767 (18)	0.0373 (9)	
H9	0.863800	−0.040498	0.883635	0.045*	
C10	0.8484 (4)	−0.2998 (7)	0.93287 (18)	0.0411 (9)	
H10	0.941490	−0.328690	0.942456	0.049*	
C11	0.7596 (4)	−0.4287 (7)	0.95435 (19)	0.0429 (9)	
H11	0.792211	−0.543911	0.978995	0.051*	
C12	0.6235 (5)	−0.3897 (7)	0.93991 (19)	0.0421 (9)	
H12	0.562923	−0.477628	0.954888	0.051*	
C13	0.5753 (4)	−0.2218 (7)	0.90350 (19)	0.0398 (9)	
H13	0.481869	−0.196631	0.892795	0.048*	
C14	0.5379 (4)	1.0742 (6)	0.59755 (16)	0.0316 (8)	
H14	0.447555	1.034414	0.574841	0.038*	
C15	0.6388 (4)	1.0822 (6)	0.55688 (16)	0.0324 (7)	
C16	0.5864 (4)	1.4325 (7)	0.57621 (16)	0.0364 (8)	
H16A	0.511318	1.499253	0.548791	0.044*	
H16B	0.647322	1.543044	0.595920	0.044*	
C17	0.5353 (4)	1.2951 (6)	0.62119 (17)	0.0380 (9)	
H17A	0.594166	1.307299	0.660417	0.046*	
H17B	0.443319	1.335774	0.624282	0.046*	

### Atomic displacement parameters (Å^2^)

**Table:**

	*U*^11^	*U*^22^	*U*^33^	*U*^12^	*U*^13^	*U*^23^
N1	0.0382 (16)	0.0309 (16)	0.0394 (17)	−0.0030 (14)	0.0036 (13)	0.0042 (14)
O1	0.0343 (12)	0.0310 (13)	0.0353 (12)	0.0018 (11)	0.0110 (10)	0.0067 (11)
C1	0.0342 (17)	0.0311 (18)	0.0353 (17)	−0.0006 (14)	0.0112 (14)	0.0014 (14)
C2	0.0353 (17)	0.0255 (16)	0.0326 (17)	0.0003 (15)	0.0082 (14)	−0.0005 (14)
O2	0.0379 (14)	0.0525 (18)	0.0542 (17)	0.0101 (14)	0.0154 (12)	0.0194 (16)
N2	0.0376 (16)	0.0315 (16)	0.0401 (16)	−0.0028 (14)	0.0054 (13)	0.0052 (14)
C3	0.0338 (18)	0.032 (2)	0.0356 (18)	−0.0040 (14)	0.0078 (14)	0.0005 (15)
N3	0.0408 (17)	0.0307 (16)	0.0383 (16)	−0.0016 (13)	0.0124 (13)	0.0019 (13)
O3	0.0593 (17)	0.0332 (14)	0.0482 (15)	0.0062 (14)	0.0266 (13)	0.0002 (13)
C4	0.0340 (17)	0.0296 (17)	0.0392 (19)	−0.0018 (15)	0.0056 (14)	0.0019 (15)
C5	0.0364 (17)	0.0278 (19)	0.0338 (17)	−0.0016 (14)	0.0064 (14)	0.0002 (14)
C6	0.0341 (17)	0.0358 (19)	0.0364 (17)	−0.0029 (16)	0.0094 (13)	0.0030 (17)
C7	0.0335 (17)	0.0322 (19)	0.0391 (18)	−0.0003 (15)	0.0109 (14)	0.0025 (16)
C8	0.0401 (18)	0.0289 (18)	0.0331 (17)	0.0007 (16)	0.0045 (14)	0.0017 (15)
C9	0.0396 (19)	0.034 (2)	0.0379 (18)	−0.0004 (16)	0.0071 (15)	0.0014 (15)
C10	0.044 (2)	0.035 (2)	0.042 (2)	0.0030 (17)	0.0034 (17)	0.0014 (17)
C11	0.052 (2)	0.0306 (19)	0.043 (2)	0.0007 (18)	0.0033 (17)	0.0040 (17)
C12	0.048 (2)	0.035 (2)	0.043 (2)	−0.0058 (18)	0.0077 (17)	0.0048 (17)
C13	0.041 (2)	0.0339 (19)	0.044 (2)	−0.0036 (16)	0.0051 (16)	0.0024 (17)
C14	0.0337 (16)	0.0301 (17)	0.0319 (16)	0.0031 (14)	0.0087 (13)	0.0032 (14)
C15	0.0356 (17)	0.0312 (17)	0.0308 (16)	0.0004 (14)	0.0080 (13)	−0.0004 (14)
C16	0.0454 (19)	0.0282 (18)	0.0367 (18)	0.0008 (17)	0.0108 (15)	0.0007 (16)
C17	0.048 (2)	0.0310 (19)	0.0374 (19)	0.0051 (16)	0.0149 (16)	−0.0018 (16)

### Geometric parameters (Å, º)

**Table:**

N1—N2	1.251 (5)	C7—H7	0.9500
N1—C5	1.431 (5)	C8—C9	1.400 (5)
O1—C1	1.347 (4)	C8—C13	1.391 (6)
O1—C14	1.440 (4)	C9—H9	0.9500
C1—C2	1.484 (5)	C9—C10	1.381 (6)
C1—O2	1.208 (5)	C10—H10	0.9500
C2—C3	1.391 (5)	C10—C11	1.387 (6)
C2—C7	1.406 (5)	C11—H11	0.9500
N2—C8	1.433 (5)	C11—C12	1.386 (7)
C3—H3	0.9500	C12—H12	0.9500
C3—C4	1.381 (5)	C12—C13	1.392 (6)
N3—H3A	0.8800	C13—H13	0.9500
N3—C15	1.336 (5)	C14—H14	1.0000
N3—C16	1.462 (5)	C14—C15	1.530 (5)
O3—C15	1.232 (5)	C14—C17	1.514 (5)
C4—H4	0.9500	C16—H16A	0.9900
C4—C5	1.391 (5)	C16—H16B	0.9900
C5—C6	1.400 (5)	C16—C17	1.532 (5)
C6—H6	0.9500	C17—H17A	0.9900
C6—C7	1.383 (6)	C17—H17B	0.9900
			
N2—N1—C5	114.3 (3)	C9—C10—H10	119.7
C1—O1—C14	115.3 (3)	C9—C10—C11	120.6 (4)
O1—C1—C2	112.4 (3)	C11—C10—H10	119.7
O2—C1—O1	123.3 (4)	C10—C11—H11	119.9
O2—C1—C2	124.3 (4)	C12—C11—C10	120.1 (4)
C3—C2—C1	122.1 (3)	C12—C11—H11	119.9
C3—C2—C7	120.1 (4)	C11—C12—H12	120.0
C7—C2—C1	117.8 (3)	C11—C12—C13	120.1 (4)
N1—N2—C8	113.6 (3)	C13—C12—H12	120.0
C2—C3—H3	120.1	C8—C13—C12	119.5 (4)
C4—C3—C2	119.8 (4)	C8—C13—H13	120.3
C4—C3—H3	120.1	C12—C13—H13	120.3
C15—N3—H3A	122.5	O1—C14—H14	110.2
C15—N3—C16	115.0 (3)	O1—C14—C15	107.8 (3)
C16—N3—H3A	122.5	O1—C14—C17	113.4 (3)
C3—C4—H4	119.8	C15—C14—H14	110.2
C3—C4—C5	120.4 (4)	C17—C14—H14	110.2
C5—C4—H4	119.8	C17—C14—C15	104.8 (3)
C4—C5—N1	116.1 (3)	N3—C15—C14	107.2 (3)
C4—C5—C6	120.0 (3)	O3—C15—N3	127.5 (4)
C6—C5—N1	123.9 (3)	O3—C15—C14	125.3 (4)
C5—C6—H6	120.1	N3—C16—H16A	111.1
C7—C6—C5	119.7 (4)	N3—C16—H16B	111.1
C7—C6—H6	120.1	N3—C16—C17	103.4 (3)
C2—C7—H7	120.1	H16A—C16—H16B	109.0
C6—C7—C2	119.9 (3)	C17—C16—H16A	111.1
C6—C7—H7	120.1	C17—C16—H16B	111.1
C9—C8—N2	123.5 (3)	C14—C17—C16	104.6 (3)
C13—C8—N2	116.0 (3)	C14—C17—H17A	110.8
C13—C8—C9	120.5 (4)	C14—C17—H17B	110.8
C8—C9—H9	120.4	C16—C17—H17A	110.8
C10—C9—C8	119.2 (4)	C16—C17—H17B	110.8
C10—C9—H9	120.4	H17A—C17—H17B	108.9
			
N1—N2—C8—C9	−6.9 (5)	C3—C4—C5—C6	−3.6 (6)
N1—N2—C8—C13	173.6 (4)	N3—C16—C17—C14	−20.1 (4)
N1—C5—C6—C7	−177.6 (4)	C4—C5—C6—C7	1.9 (6)
O1—C1—C2—C3	12.1 (5)	C5—N1—N2—C8	178.4 (3)
O1—C1—C2—C7	−164.8 (3)	C5—C6—C7—C2	0.8 (6)
O1—C14—C15—N3	−137.4 (3)	C7—C2—C3—C4	0.1 (6)
O1—C14—C15—O3	44.1 (5)	C8—C9—C10—C11	−1.2 (6)
O1—C14—C17—C16	139.4 (3)	C9—C8—C13—C12	1.2 (6)
C1—O1—C14—C15	−163.5 (3)	C9—C10—C11—C12	0.9 (6)
C1—O1—C14—C17	81.0 (4)	C10—C11—C12—C13	0.4 (6)
C1—C2—C3—C4	−176.7 (3)	C11—C12—C13—C8	−1.5 (6)
C1—C2—C7—C6	175.1 (4)	C13—C8—C9—C10	0.1 (6)
C2—C3—C4—C5	2.6 (6)	C14—O1—C1—C2	179.2 (3)
O2—C1—C2—C3	−168.9 (4)	C14—O1—C1—O2	0.2 (6)
O2—C1—C2—C7	14.2 (6)	C15—N3—C16—C17	10.8 (4)
N2—N1—C5—C4	−170.8 (3)	C15—C14—C17—C16	22.2 (4)
N2—N1—C5—C6	8.6 (5)	C16—N3—C15—O3	−178.1 (4)
N2—C8—C9—C10	−179.4 (4)	C16—N3—C15—C14	3.4 (4)
N2—C8—C13—C12	−179.2 (4)	C17—C14—C15—N3	−16.3 (4)
C3—C2—C7—C6	−1.8 (6)	C17—C14—C15—O3	165.1 (4)
C3—C4—C5—N1	175.9 (3)		

### Hydrogen-bond geometry (Å, º)

*Cg*3 is the centrod of the C8–C13 ring.

**Table:**

*D*—H···*A*	*D*—H	H···*A*	*D*···*A*	*D*—H···*A*
N3—H3*A*···O3^i^	0.88	1.99	2.868 (4)	175
C11—H11···*Cg*3^ii^	0.95	2.76	3.596 (5)	147

Symmetry codes: (i) −*x*+3/2, *y*+1/2, −*z*+1; (ii) −*x*+3/2, *y*−1/2, −*z*+2.
